# Acid bone lysates reduce bone regeneration in rat calvaria defects

**DOI:** 10.1002/jbm.a.37050

**Published:** 2020-07-10

**Authors:** Franz‐Josef Strauss, Ulrike Kuchler, Reiko Kobatake, Patrick Heimel, Stefan Tangl, Reinhard Gruber

**Affiliations:** ^1^ Department of Oral Biology School of Dentistry, Medical University of Vienna Vienna Austria; ^2^ Department of Conservative Dentistry Faculty of Dentistry, University of Chile Santiago Chile; ^3^ Clinic of Reconstructive Dentistry, Center of Dental Medicine University of Zurich Zurich Switzerland; ^4^ Department of Oral Surgery School of Dentistry, Medical University of Vienna Vienna Austria; ^5^ Department of Advanced Prosthodontics Hiroshima University Higashihiroshima Japan; ^6^ Ludwig Boltzmann Institute for Experimental and Clinical Traumatology Vienna Austria; ^7^ Core Facility Hard Tissue and Biomaterial Research Karl Donath Laboratory, School of Dentistry, Medical University of Vienna Vienna Austria; ^8^ Austrian Cluster for Tissue Regeneration Vienna Austria; ^9^ Department of Periodontology School of Dental Medicine, University of Bern Bern Switzerland

**Keywords:** bone allograft, bone grafts, bone regeneration, growth factors

## Abstract

Acid bone lysates (ABLs) represent the growth factors and other molecules released during autologous graft resorption. However, the impact of these bone‐derived growth factors on the healing of bone defects has not yet been investigated. The aim of the present study was, therefore, to examine the impact of ABLs adsorbed to collagen membranes on bone regeneration. To this end, in 16 female Sprague Dawley rats, a standardized 5‐mm‐diameter critical size defect on the calvarial bone was created. The defects were covered with collagen membranes that had been soaked either in serum‐free media or ABLs followed by lyophilization. After a healing period of 4 weeks, micro‐computed tomography (μCT) and histological analyses by means of undecalcified thin ground sections were performed. μCT analysis of the inner 4 mm of the calvaria defect showed a greater bone defect coverage in the control group when compared to ABL group, 29.8% (confidence interval [CI]: 17.7–50.3) versus 5.6% (CI: 1.0–29.8, *p* = .03), respectively. Moreover, we found significantly more absolute bone volume (BV) in the control group when compared to ABL group, 0.59 mm^3^ (CI: 0.27–1.25) versus 0.07 mm^3^ (CI: 0.06–0.59, *p* = .04), respectively. Histomorphometry confirmed these findings with a relative BV in the central compartment of 14.1% (CI: 8.4–20.6) versus 5.6% (CI: 3.4–7.9, *p* = .004), respectively. These findings indicate that bone‐derived growth factors contained in ABLs are able to attenuate bone regeneration within collagen membranes.

## 1 INTRODUCTION

Bone augmentation has become a standard procedure driven by the need to increase bone volume (BV) in atrophic jaws prior to implant placement. One common procedure for bone augmentation is the use of autologous bone grafts, either as bone blocks or as bone chips (Naenni, Lim, Papageorgiou, & Hammerle, 2019). Autologous bone grafts, however, undergo resorption leading to the shrinkage of the bone block or the disappearance of the bone chips (Naenni et al., 2019). Up to one‐quarter of the original, graft size can be resorbed at the time of implant placement (Mertens et al., 2013). This osteoclastic resorption occurs during the early graft consolidation process at the augmented site (Saulacic et al., 2015). Despite its susceptibility to catabolic changes, autologous bone is still considered the gold standard for bone augmentation, mainly due to the enhanced performance compared to bone substitutes during the early phases of bone healing (Buser et al., 1998). Apart from these favorable osteoconductive properties of autografts, it is widely believed that the growth factors released by osteoclasts during graft resorption, including TGF‐β, support the process of bone regeneration.

TGF‐β is one of the most abundant growth factors in the bone matrix (Bismar et al., 1999) which can be released during bone resorption or under acidic conditions (Hauschka, Mavrakos, Iafrati, Doleman, & Klagsbrun, 1986; Pfeilschifter et al., 1998; Strauss et al., 2018). TGF‐β is then capable of binding to dental implants and collagen membranes (Stahli, Miron, Bosshardt, Sculean, & Gruber, 2016; Strauss et al., 2019). Nevertheless, the role of TGF‐β is not so clear, as it can support but also hinder bone regeneration. For example, bone‐derived TGF‐β recruits mesenchymal cells, the progenitors of bone‐forming osteoblasts, to the site of bone remodeling (Crane & Cao, 2014). Similarly, TGF‐β initiates and promotes heterotopic ossification in mice via recruiting mesenchymal progenitors (Wang et al., 2018). TGF‐β1 induces bone closure of rabbit skull defects (Beck et al., 1993) and TGF‐β1‐loaded implants provoke an increased bone surface area in a rabbit cranial defect model (Vehof, Haus, de Ruijter, Spauwen, & Jansen, 2002). In contrast, TGF‐β1 loaded β‐TCP failed to support bone formation in a rat calvaria model (Elimelech et al., 2019) and in vitro, TGF‐β reduces osteogenic differentiation in cell culture models (Noda & Rodan, 1986). High‐doses of TGF‐β1 also dampened bone regeneration by repressing the bone morphogenetic protein 2 activity (Xu et al., 2020). Therefore, and considering that acid bone lysates (ABLs) are a rich source of TGF‐β that binds to collagen (Strauss et al., 2018), the question arises whether the local application of ABLs supports or hinders bone regeneration.

Recently, we characterized the molecular composition of ABLs prepared from porcine bone chips (Strauss et al., 2018). We showed that activation of the TGF‐β signaling pathway is the major response of mesenchymal cells upon their exposure to ABLs along with revealing the expected decrease of in vitro osteogenic differentiation (Strauss et al., 2018). Since TGF‐β adsorbs to collagen (Hempel et al., 2012) and thus to the classical collagen barrier membranes (Stahli et al., 2016), these membranes can be considered as suitable carriers to investigate ABLs on bone regeneration in vivo. Using this approach, we recently demonstrated that bone‐conditioned medium lyophilized onto collagen membranes slightly reduces bone formation in rat calvaria defects (Kuchler et al., 2018). Taking advantage of this established model, the aim of the present study was, therefore, to examine the impact of ABL adsorbed to collagen membranes on bone regeneration in rat calvaria defects.

## 2 MATERIAL AND METHODS

### 2.1 Study design

The present study was conducted at the Department of Biomedical Research of the Medical University of Vienna following the ARRIVE guidelines (Kilkenny, Browne, Cuthill, Emerson, & Altman, 2011). Before starting the study, an approval of the study protocol was obtained by the local ethical committee at the Medical University of Vienna (GZ BMWFW‐66.009/0217‐WF/V/3b/2015). Briefly, sixteen 7‐month old (200–300 g) female Sprague Dawley rats from the Division for Biomedical Research (Himberg, Austria) were randomly allocated into two groups with eight animals each: control group received collagen membranes soaked in serum‐free medium (SFM) and ABLs group received collagen membranes soaked in ABLs. Collagen membranes (25 mm × 25 mm; Bio‐Gide®, Geistlich, Wolhusen, Switzerland) were loaded with SFM or with 1 ml of pooled ABLs and frozen at −80°C. Lyophilization was then performed using a freeze dryer Alpha 1–2 LDplus (Martin Christ, Osterode am Harz, Germany). Randomization was performed via a computer‐generated randomization. The animals were treated according to the guidelines for animal care with free access to water and a standard diet (Kilkenny, Browne, Cuthill, Emerson, & Altman, 2010).

### 2.2 Acid bone lysate

ABL was prepared as recently described (Strauss et al., 2018; Strauss et al., 2019). Bone was obtained from adult pigs within 6 hr post‐mortem (Fleischerei Leopold Hödl, Vienna, Austria). Bone chips from the mandible, calvaria, and tibia were harvested with a bone scraper (Hu‐Friedy, Rotterdam, The Netherlands). Thereafter, the bone chips were cleaned using Dulbecco's modified Eagle medium that was supplemented with antibiotics (Invitrogen Corporation, Carlsbad, CA). Five grams of wet bone chips were incubated while being stirred with 50 ml of 0.1 N HCl (10% weight/volume) at room temperature. ABLs were harvested after 16 hr, centrifuged, and then pH neutralized. Subsequently, another centrifugation was performed. ABLs were then filtered sterile using a 0.2 μm syringe filter (VWR International, PA) and kept frozen at −80°C. Right before each experiment, the stocks were thawed.

### 2.3 Surgical procedures and postoperative treatment

The surgical procedures were performed as previously described (Kuchler et al., 2018). Briefly, all rats received ketamine (50 mg/kg i.p.) (AniMedica, Senden, Erlangen, Germany) and xylazine hydrochloride (10 mg/kg i.p) (Bayer Austria, Vienna, Austria). A standardized 5‐mm‐diameter critical size defect was created on the calvaria bone by the use of a trephine burr (Medos Medizintechnik; Vienna, Austria). The collagen membrane was trimmed and placed onto the defects. The membrane overlapped the walls of the defect by at least 1 mm. Thereafter, the membrane was stabilized and the flap was sutured in layers with resorbable sutures (Vicryl 5‐0; Ethicon GmbH, Norderstedt, Germany). Buprenorphine 0.06 mg/kg, (Temgesic®, Temgesic, Reckitt, and Colman Pharm., Hull, UK) and piritramide in drinking water ad lib was administered for pain relief. After 4 weeks of healing, animals were sacrificed by an intracardial overdose of sodium pentobarbital (300 mg/kg). Samples from each calvarium was obtained and further processed for micro‐computed tomographic (μCT) and histological analysis.

### 2.4 μCT analysis

After euthanasia, the 16 heads were fixed in phosphate‐buffered formalin (Roti‐Histofix 4%, Carl Roth, Karlsruhe, Germany). μCT was carried out at 90 kV/200 μA with an isotropic resolution of 17.2 μm and an integration time of 500 ms (μCT 50 Scanco Medical AG, Bruttisellen, Switzerland). The images were rotated using Amira 6.2 (Thermo Fisher Scientific, Waltham, MA) to obtain the drill direction in the Z axis with the defect near the center of the image. Via the Definiens Developer XD2® software (Munich, Germany, Version 2.1.1), the region of interest (ROI) was segmented from the μCT images with an individually developed ruleset. The mineralized tissue within the inner 4 mm of the defect was measured.

### 2.5 Histological and histomorphometric analysis

The 16 samples were dehydrated with ascending alcohol grades and embedded in light‐curing resin (Technovit 7200 VLC + BPO; Kulzer & Co., Wehrheim, Germany). Blocks were further processed using EXAKT cutting and grinding equipment (Exakt Apparatebau, Norderstedt, Germany). Thin‐ground sections from all samples were prepared in a plane parallel to the sagittal suture and through the center of the defect and stained with Levai–Laczko dye. The slices were scanned using an Olympus BX61VS digital virtual microscopy system (DotSlide 2.4, Olympus, Japan, Tokyo) with a 20× objective resulting in a resolution of 0.32 μm per pixel and then quantified using Adobe Photoshop® software (Adobe, San Jose, CA). Histomorphometric analysis was performed at three ROIs representing (a) the central compartment within the defect margins, (b) the adjacent ectocranial compartments, and (c) the outer compartment on the surface of the host's cortical bone.

### 2.6 Statistics

Statistical analysis was based on the data observed with the μCT and histomorphometric analysis. The Shapiro–Wilk test was used to test the normality of the data sets. For μCT, median values and confidence intervals (CIs) of the primary outcome (percentage of bone defect coverage) and the BV between control and test group were compared with Mann–Whitney *U* test due to the distribution of the data. For histomorphometry, BV per tissue volume (BV/TV in %) between control and test group were compared with Mann–Whitney U test. Analyses were performed using Prism v7 (GraphPad, La Jolla, CA). Owing to the pilot nature of the study, the sample size was chosen based on experience from previous studies (Kuchler et al., 2018) to balance the ability to measure significant differences while reducing the number of animals used. Significance was set at *p* < .05.

## 3 RESULTS

### 3.1 μCT analysis

**FIGURE 1:**
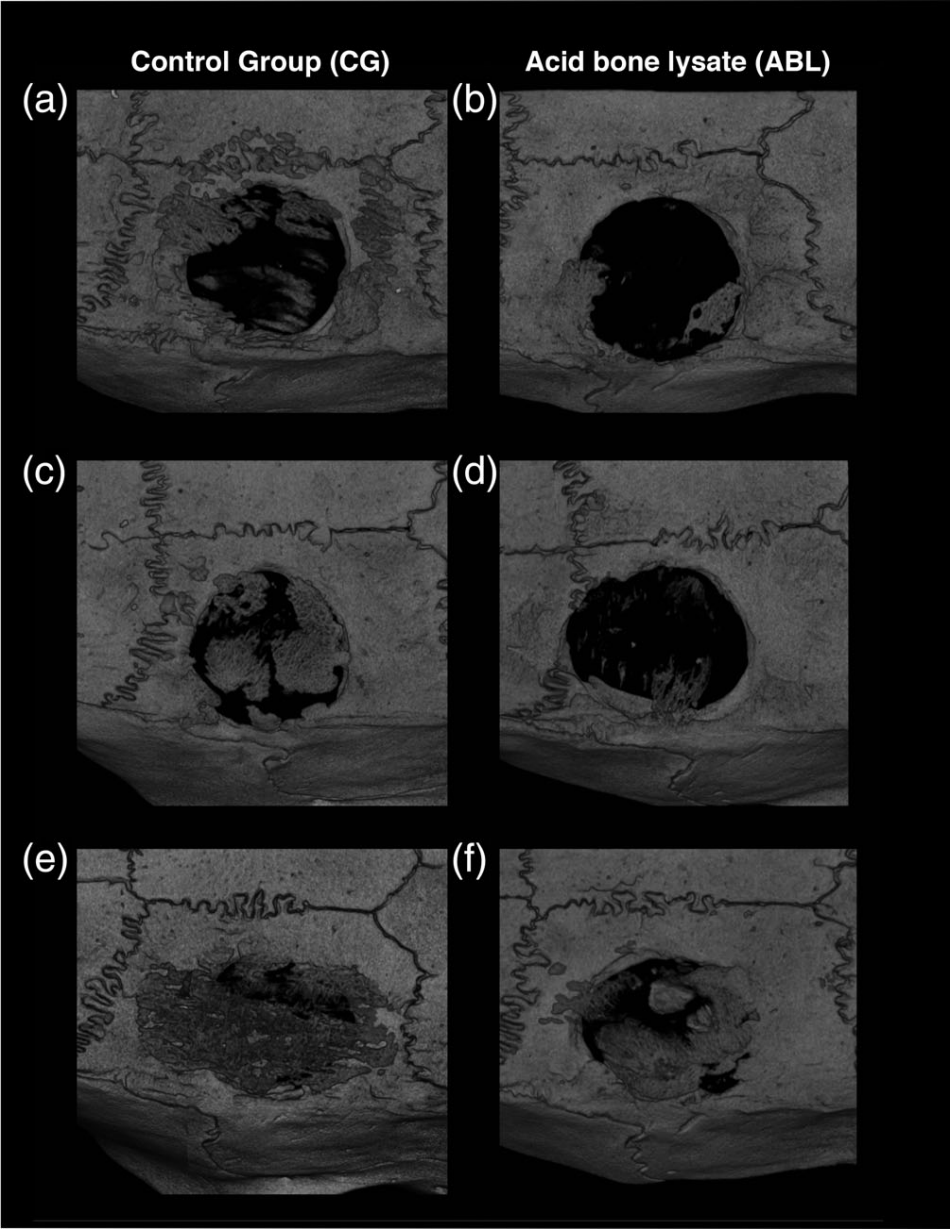
Micro‐CT overview of the defect anatomy, bone in contact with host bone and bony islands after 4 weeks of healing. Rat calvaria defects were treated with collagen membranes either soaked in (a, c, e) serum‐free medium, or (b, d, f) in acid bone lysates (ABL). Micro‐CT pictures representing the samples with (a, b) minimum, (c, d) median, and (e, f) maximum bone volume based on quantitative analysis

**FIGURE 2:**
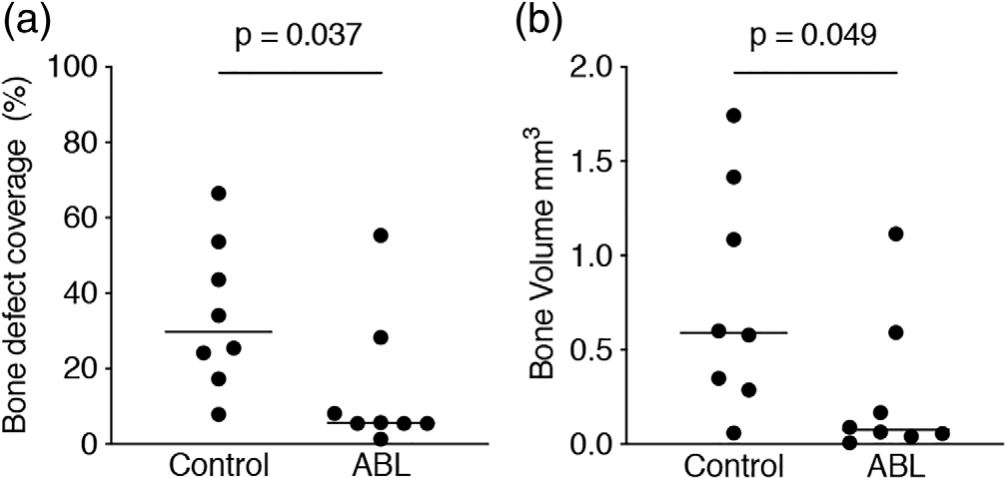
Acid bone lysate (ABL) reduces bone regeneration in the calvaria defect. Quantitative analysis of (a) bone defect coverage and (b) bone volume in the region of interest (the inner 4 mm diameter of a 5 mm defect). Statistical analysis was based on the data observed with the μCT analysis. The two groups were compared with Mann–Whitney *U* test. *p*‐values are indicated

Figure 1 shows three representative samples per group of the calvaria defect, corresponding to the minimum (a, b), median (c, d), and maximum (e, f) value in terms of bone regeneration. The control group displayed higher amounts of BV compared to the ABL group. Moreover, there was a subjective impression of a bone formation pattern, possibly caused by the fibrils of the collagen membrane (Figure 1). Quantitative analysis of the inner 4 mm of the defect showed that the relative bone area was significantly higher in the control group than in the collagen membranes soaked and lyophilized with ABLs, 29.8% (CI: 17.7–50.3) versus 5.6% (CI: 1.0–29.8), respectively (*p* = .03) (Figure 2a). Quantitative analysis further displayed that also the BV was significantly higher in the control group compared to the ABL group, 0.59 mm^3^ (CI: 0.27–1.25) versus 0.07 mm^3^ (CI: 0.06–0.59), respectively (*p* = .04) (Figure 2b). Taken together, these findings suggest that ABLs lyophilized onto collagen membranes reduce bone formation in a rat calvarial defect model.

### 3.2 Histomorphometric analysis

**FIGURE 3:**
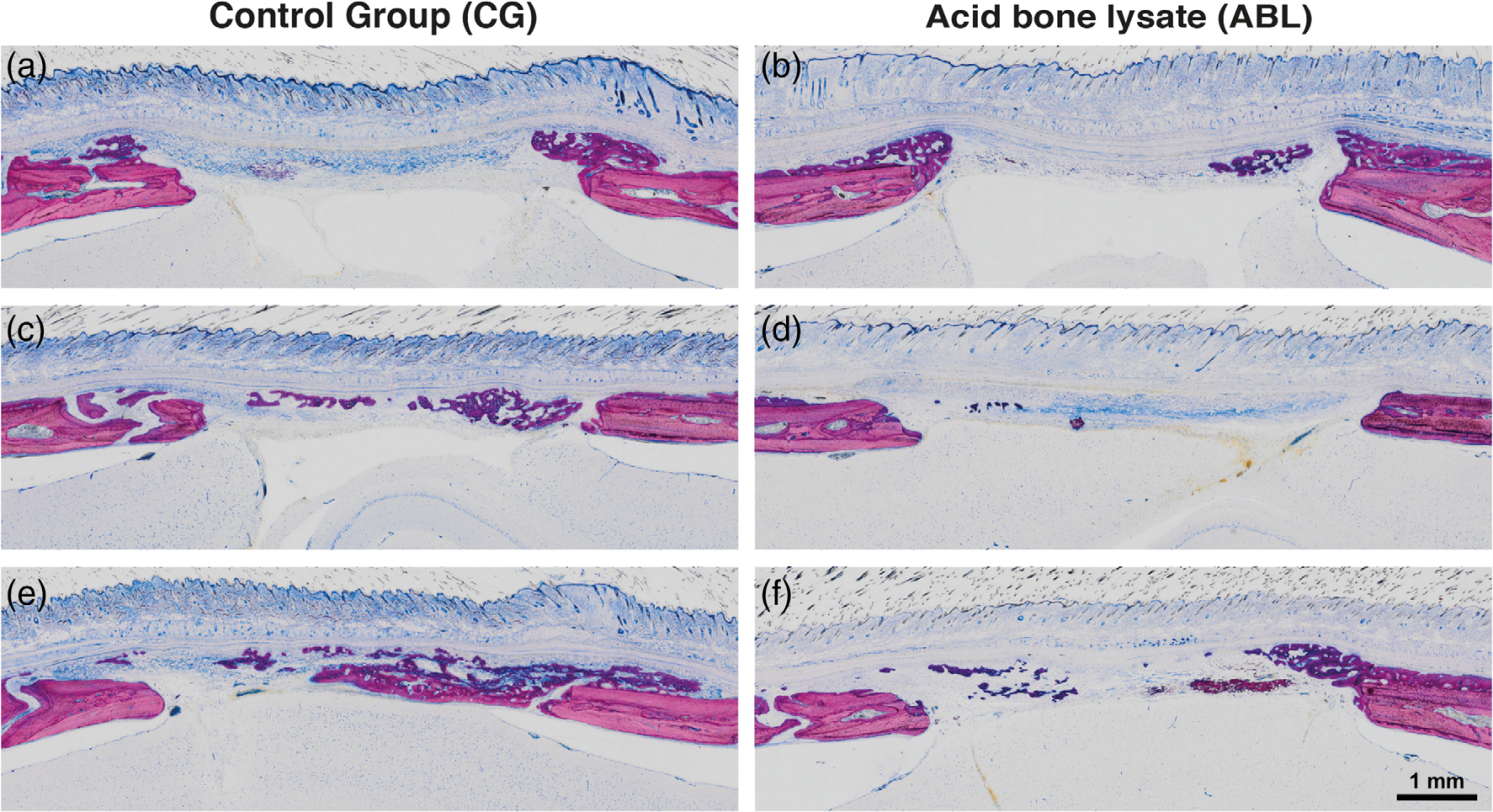
Histological overview of the defect anatomy after 4 weeks of healing. Rat calvaria defects were treated with native collagen membranes either soaked in serum‐free medium (a, c, e), or in acid bone lysate (ABL) (b, d, f). Histological pictures representing the samples with minimum (a, b), median (c, d), and maximum (e, f) bone volume based on quantitative analysis. The local host calvaria bone demarcates the defect borders and appears in light purple. The newly formed bone stained in dark purple appears in the spongy part of the collagen membranes

**FIGURE 4:**
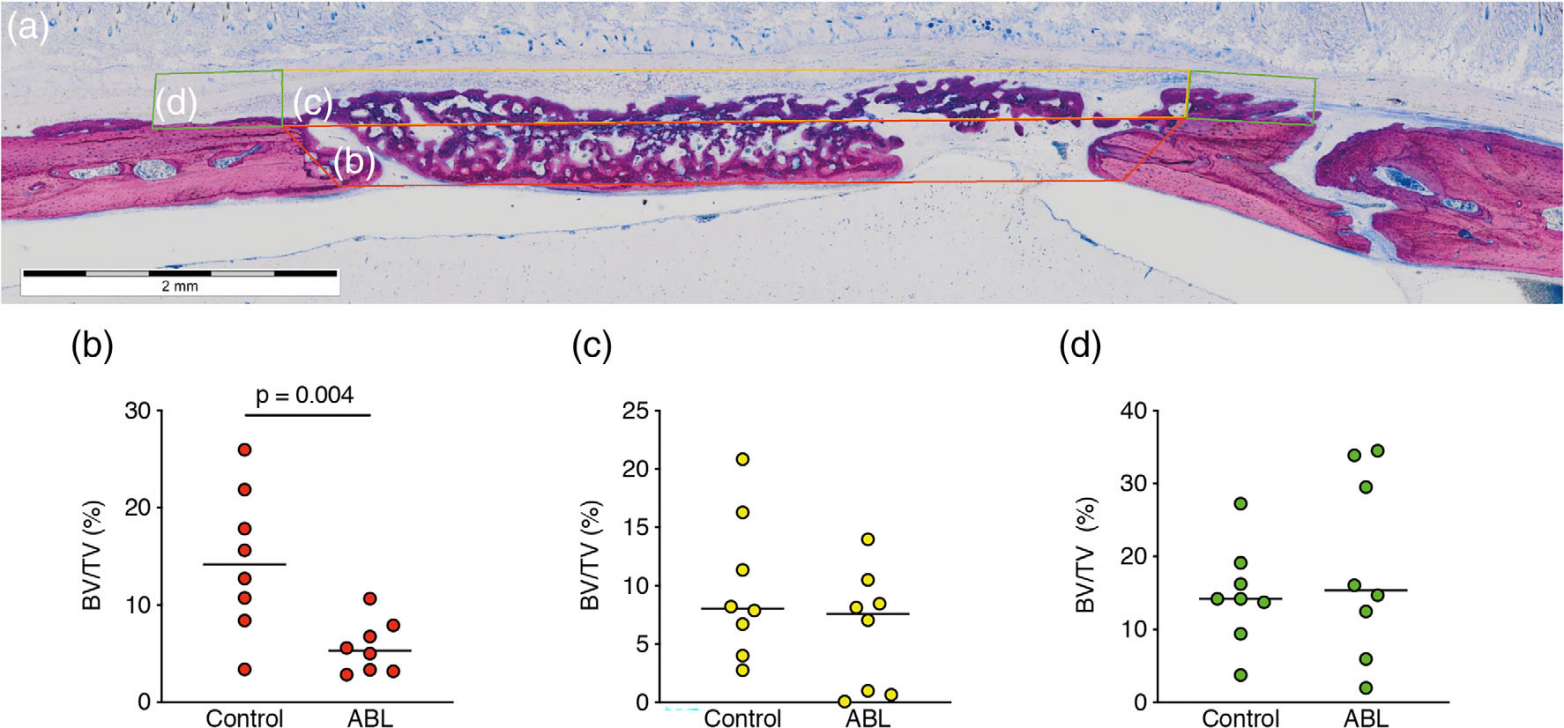
The effect of acid bone lysates is restricted to the central compartment. (a) Histomorphometry on bone volume per tissue volume BV/TV (%) was performed at three regions of interest (ROI); (b, Red ROI) the central compartment within defect margins, (c, yellow ROI) the adjacent ectocranial compartments, and (d, green ROI) the outer compartment on the surface of the host cortical bone. The groups were compared with Mann–Whitney U test. *p*‐values are indicated

Figure 3 shows three representative ground sections per group of the calvaria defect, corresponding to the minimum (a, b), median (c, d), and maximum (e, f) values in terms of bone regeneration according to the μCT analysis. In agreement with the μCT analysis, the control group showed more bone formation as compared to ABL group. To confirm these subjective impressions, an histomorphometric analysis was conducted (Figure 4a). The histomorphometric analysis revealed that in the central compartment of the defect, the percentage of BV per tissue volume (BV/TV) was significantly higher in the control group compared to ABL group, 14.1% (CI: 8.4–20.6) versus 5.6% (CI: 3.4–7.9, *p* = .004), respectively (Figure 4b). Next, and in order to determine whether the effect of ABL was restricted to the central compartment, two other ROIs were analyzed. The percentage of BV/TV did not differ between the control and ABL group, neither in the ectocranial compartments of the defect, 8.0% (CI: 4.6–14.9) versus 7.5% (CI: 1.9–10.5, *p* > .05) (Figure 4c), nor in the outer compartment encompassing the external surface of the defect's margin, 14.2% (CI: 9.0–20.4) versus 15.4% (CI: 8.1–29.13, *p* > .05), respectively (Figure 4d). Overall, these observations indicate that the effects of ABLs are restricted to the area encompassed by the collagen membrane.

### 3.3 Histological analysis

**FIGURE 5:**
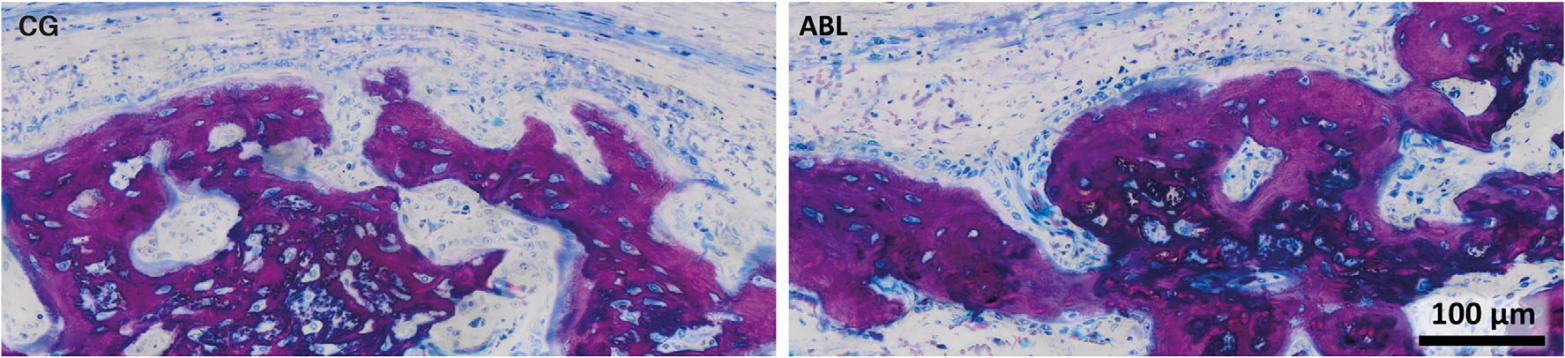
Detailed overview on the new bone in the control group (CG) and in the acid bone lysate (ABL) group. Note the characteristic features of immature woven bone indicated by the intense purple stain and the large osteocyte lacunae. The dense art of the membrane is visible in the upper part of control group also showing that new bone grows on the spongy part of the collagen membrane

Histological analysis confirmed previous findings that bone formation mainly occurs inside the collagen membrane (Figure 5) (Kuchler et al., 2018). The fibers of the original collagen membrane (light pink) are either surrounded by the new bone or soft tissue. The calvarial defect in the control group was mainly filled by woven bone (dark purple). This woven bone formed trabecular ridges with random orientation and was enclosed either by thin layers of parallel‐fibered bone (light purple) or thin layers of osteoid. These observations together with the histomorphometric analysis suggest that the effects of ABLs on bone regeneration are restricted to the area of the collagen membrane.

## 4 DISCUSSION

The main finding of the present study was that ABLs lyophilized on collagen membranes reduce bone formation in a critical size defect on rats. Despite the high variation within each group, the impact of ABL compared to a SFM control was consistent and significant. This observation was somehow unexpected based on the widely held belief that growth factors released by osteoclast during graft resorption support bone regeneration. Certainly, acid treatment of bone chips cannot simulate the complex activity of osteoclasts via a continuous acidification and a simultaneous proteolytic cleavage of the collagen‐rich extracellular matrix by the cathepsin K and other proteases (Teitelbaum, 2000). ABLs can only partially, if at all, represent the osteoclastic activity during the early stage of graft resorption. Nevertheless, there are functional similarities between the ABL and what is released from osteoclasts. For example, in vivo, TGF‐β liberated by osteoclasts recruits mesenchymal cells to the site of bone remodeling (Crane & Cao, 2014). ABL, apart from being a rich source of TGF‐β, can activate the respective signaling pathways on mesenchymal cells (Strauss et al., 2019). It should be noted, however, that the proposed involvement of TGF‐β signaling regarding the attenuating effects of ABLs remains to be examined, for example, by using a pharmacologic inhibition of TGF‐β receptor type I kinase such as SB431542 (Inman et al., 2002).

These findings with ABLs are partially consistent with our earlier observation gathered with bone conditioned medium, which is also a bone‐derived aqueous fraction containing TGF‐β1 (Peng et al., 2015), leading to a slight reduction of bone formation in a rat calvaria defect model (Kuchler et al., 2018). Since bone conditioned medium is not subjected to acid lysis (Peng et al., 2015), this previous report may be considered a related control experiment (Kuchler et al., 2018). In vitro, TGF‐β signaling dramatically inhibits the BMP‐2‐dependent calcification (Kawahara et al., 2015), similar to what we have observed with the platelet secretome (Gruber, Kandler, Fischer, & Watzek, 2006). The present observations are supported by other reports showing that the canonical TGF‐β1 signaling, via smad‐3, decreases wound healing in mouse models (Ashcroft et al., 1999). This also holds true for fracture healing (Kawakatsu et al., 2011). In contrast, non‐canonical signaling via TGF‐β‐activated kinase 1 (TAK1) supports cutaneous tissue repair (Guo, Hutchenreuther, Carter, & Leask, 2013). In this context, it should be mentioned that ABL is a complex cocktail of 394 proteins, including but not limited to TGF‐β1 (Strauss et al., 2018), therefore possible explanations of the findings presented here should not be limited to TGF‐β signaling. Notably, bone regeneration is almost restricted to the spongy part of the collagen membrane (Kuchler et al., 2018). Again, one might speculate that bone‐derived TGF‐β tends to accumulate onto the collagen matrix increasing its concentration thereby attenuating bone formation.

When considering the histologic section and μCT, some of the newly formed bone was located outside of the defect margin and within the space created by membrane and host bone. This has raised the question whether this bone formation is independent of the presence of ABL. Apart from the central compartment, where bone regeneration was significantly advanced in the control group compared to ABL group, two other ROIs were analyzed, the ectocranial compartment and the outer compartment. Interestingly, ABL had no impact on bone formation in these two regions. It can thus be assumed that the impact of ABL is restricted to the area defined by the collagen membrane that adsorbs TGF‐β1 and other molecules serving as an osteoconductive carrier with a retard function. This osteoconductive function is supported by the regeneration pattern displayed on the μCT images suggesting that collagen membranes are not just passive barriers (Omar, Elgali, Dahlin, & Thomsen, 2019). However, it cannot be ruled out that ABLs may have modified the structure of the original collagen membrane, particularly after lyophilization and thus having an impact on bone regeneration. From these observations, it becomes crucial to determine which proteins and other molecules within the ABL remain adsorbed to the collagen membranes and are limiting bone regeneration.

The clinical relevance of the present investigation is a matter of speculation, but it provides at least a possible explanation why during the resorption phase of graft consolidation bone formation is attenuated (Saulacic et al., 2015). An abundance of TGF‐β in the local microenvironment may reduce the migration of mesenchymal cells for coupled bone formation (Xu et al., 2018). At physiological levels, however, TGF‐β1 might trigger the local expression BMP‐2 thereby promoting osteogenic differentiation. Some support for this hypothesis comes from our findings that ABLs support the expression of BMP‐2 in mesenchymal cells (Strauss et al., 2019). Based on this theory, it is not the TGF‐β released from bone by osteoclasts that initiates osteogenic differentiation, but rather an indirect effect that involves the local expression of BMP‐2. Another related aspect is the fact that demineralized bone is osteoinductive, whereas the ABL has the opposite effect. In this context, what does demineralized matrix retain that promotes bone formation contrary to the suppression in osteogenic differentiation induced by ABLs? One may speculate that the osteoinductive BMPs remain attached to the original bone extracellular matrix and are not released by the acid treatment (Wang et al., 1988). Indeed, proteomic analysis of ABLs failed to detect BMPs (Strauss et al., 2018). From a clinical point of view, it is relevant to understand how autograft resorption is coupled with bone regeneration and how the process is controlled. The present findings might become clinically relevant once it can be demonstrated that the growth factors released from bone during resorption of grafts indeed reduce or even increase bone formation.

The present study has other limitations that should be taken into consideration. First, the present report involved a xenogenic setting, using a porcine peritoneum‐derived collagen membrane and porcine ABL tested on a rat defect model. Second, the collagen membranes underwent lyophilization together with a SFM or the ABLs. Considering that we did not include a regular collagen membrane, care should be taken when interpreting the results regarding the osteoconductive properties of the original collagen membrane. Since TGF‐β and presumably also other growth factors adsorb to collagen membranes (Stahli et al., 2016), lyophilization may be avoided in future research. Potential research could focus on the question whether ABLs reduce the formation of h‐type endothelial cells that carry the osteogenic progenitors into the defect (Sivaraj & Adams, 2016), or if the migration, proliferation, or osteogenic differentiation of osteogenic cells is impaired in vivo. Research is also required to understand which of the numerous proteins in ABLs are responsible for the decrease in bone regeneration and whether the effects of ABLs only occur when loaded onto collagen membranes. The present investigation certainly provides a few answers but raises many other new questions.

Taken together, these findings indicate that bone‐derived growth factors comprised in ABLs are able to attenuate bone regeneration in a rat calvaria defect model.
